# Supplementary figures and images for: Embedded-deep-learning-based sample-to-answer device for on-site malaria diagnosis
Source: Front Bioeng Biotechnol. 2024 Jul 19;12:1392269. doi: 10.3389/fbioe.2024.1392269 (PMC11294195; doi:10.3389/fbioe.2024.1392269)

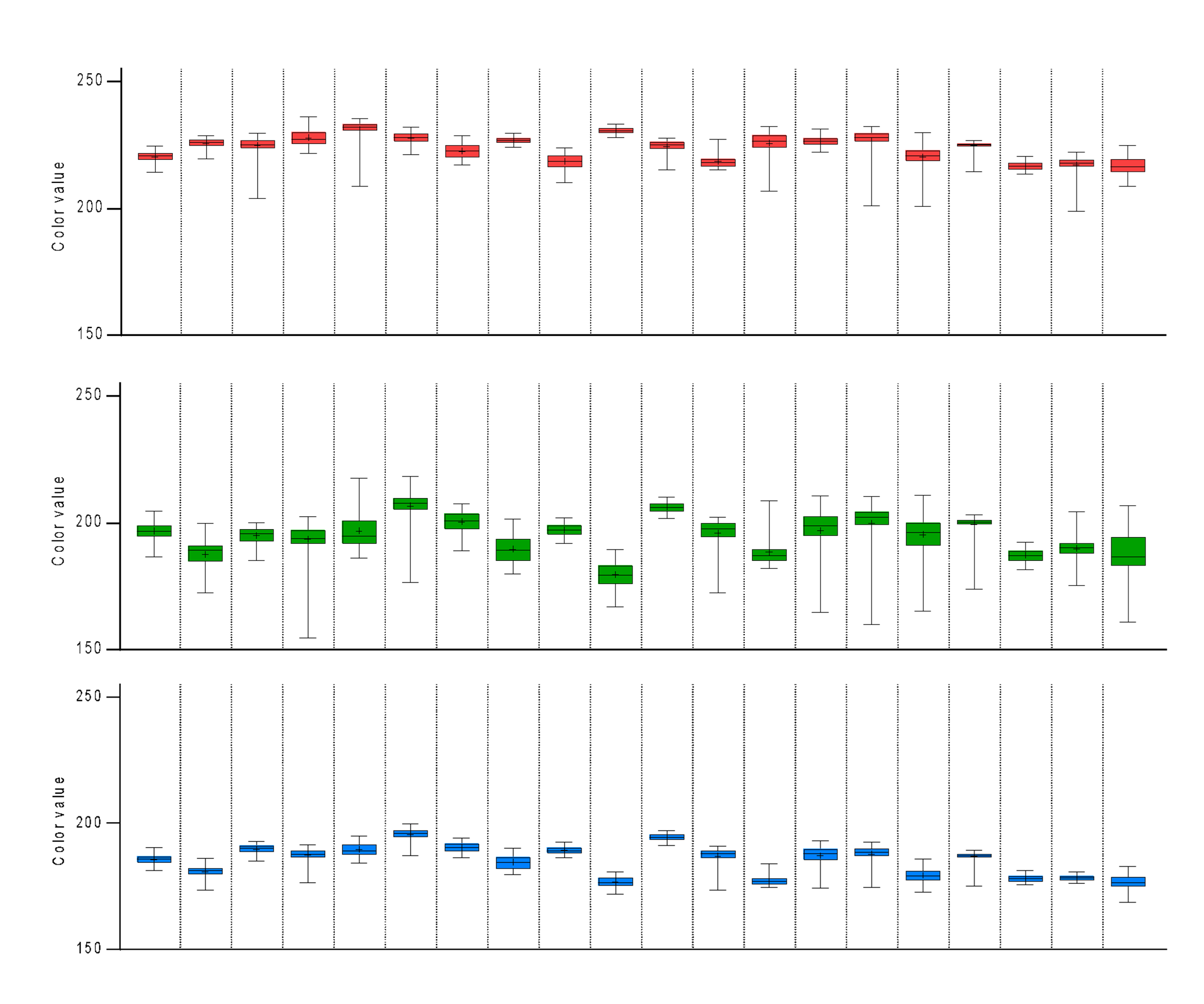

Supplement: Supplementary file 1 [file Image6.TIF]

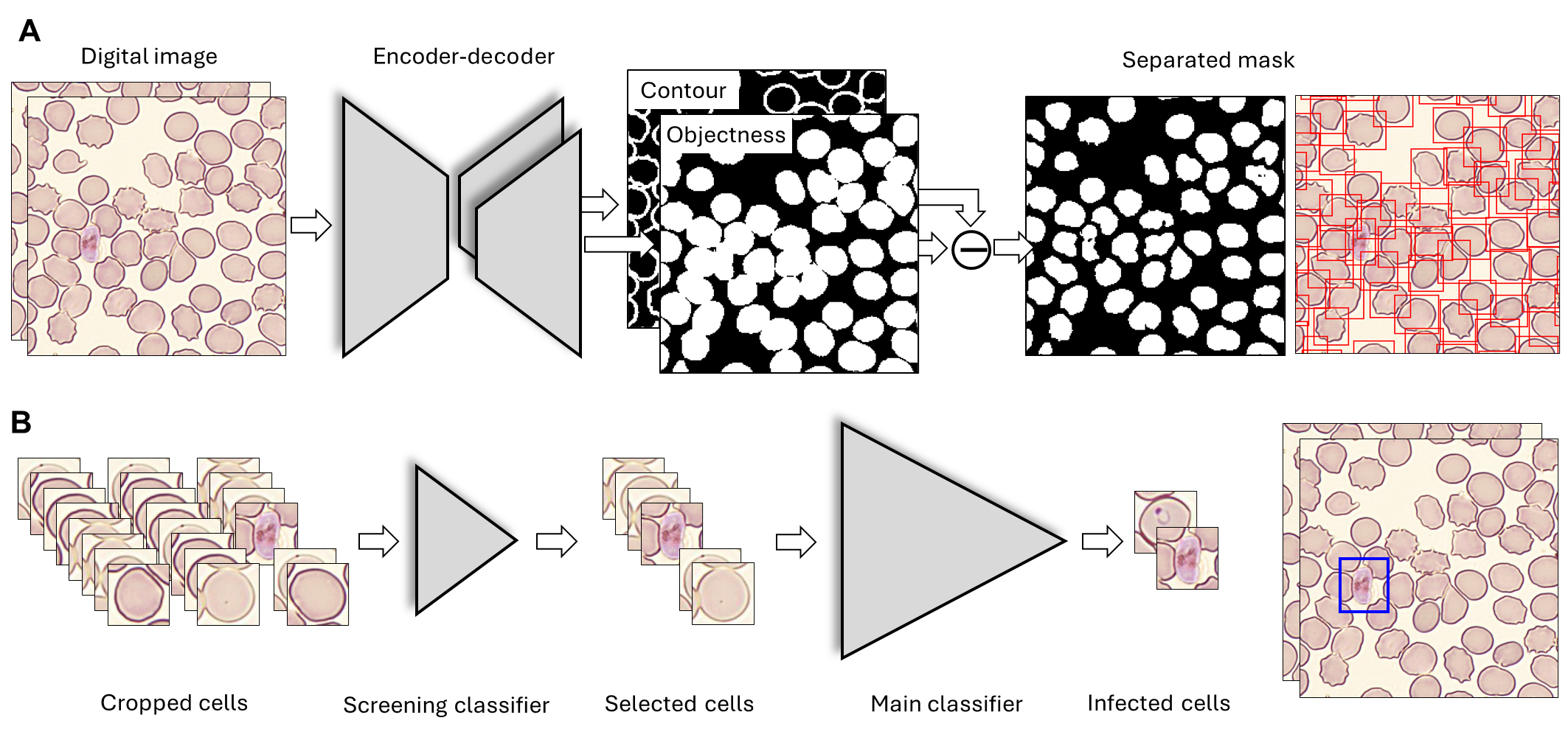

Supplement: Supplementary file 2 [file Image3.TIF]

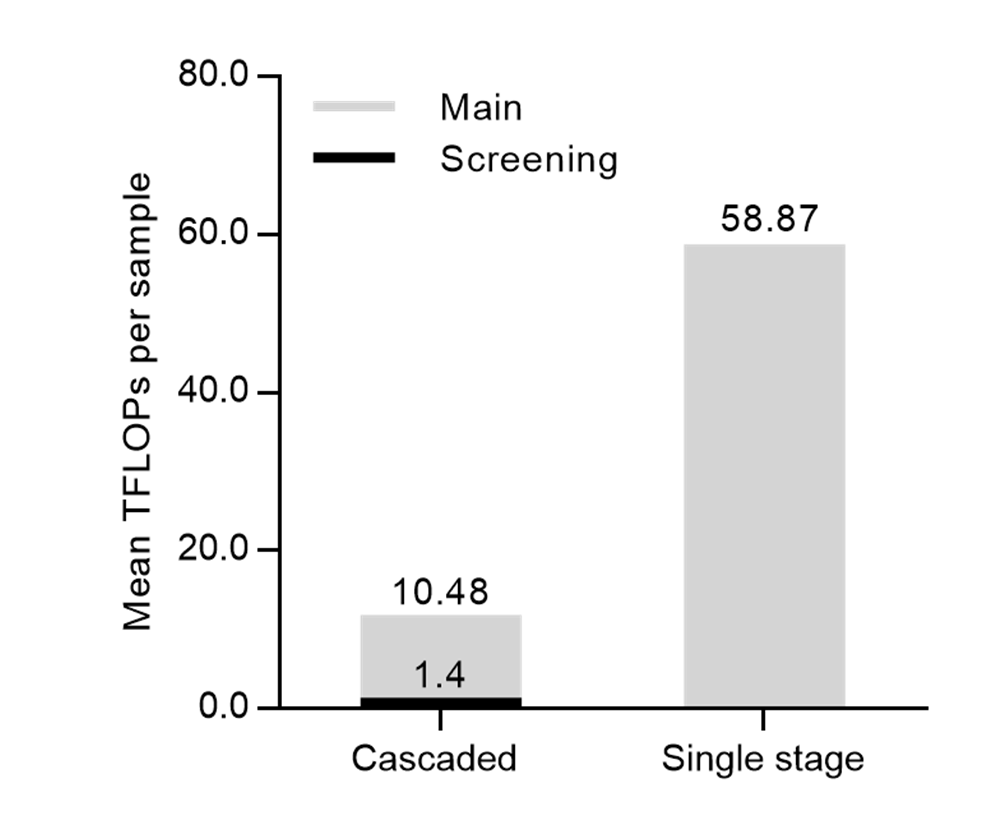

Supplement: Supplementary file 3 [file Image4.TIF]

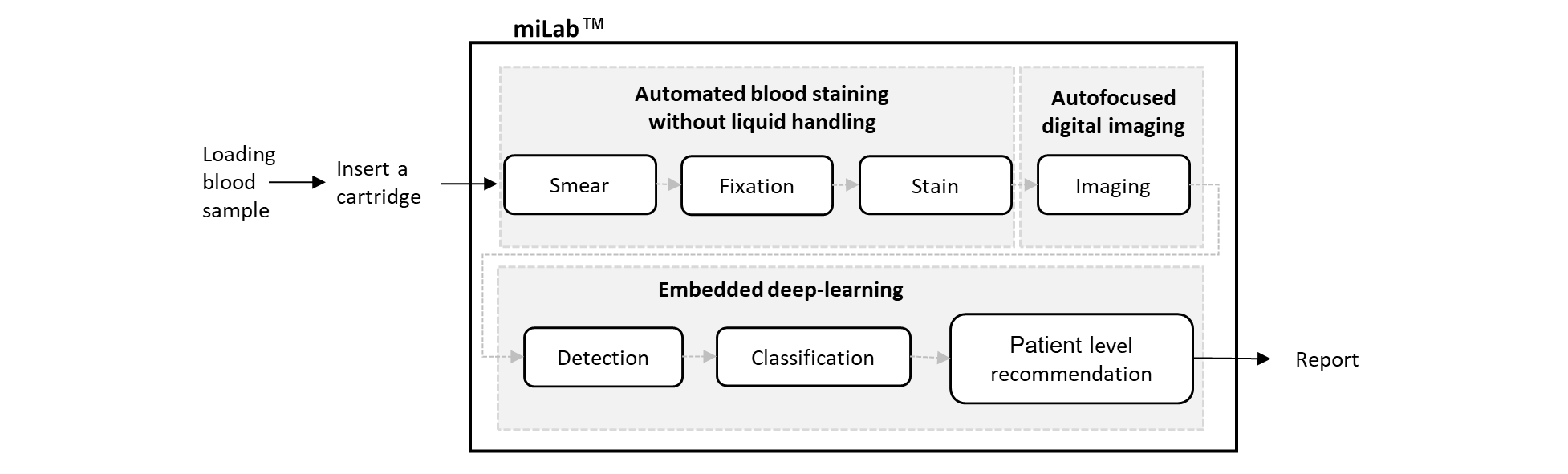

Supplement: Supplementary file 4 [file Image2.TIF]

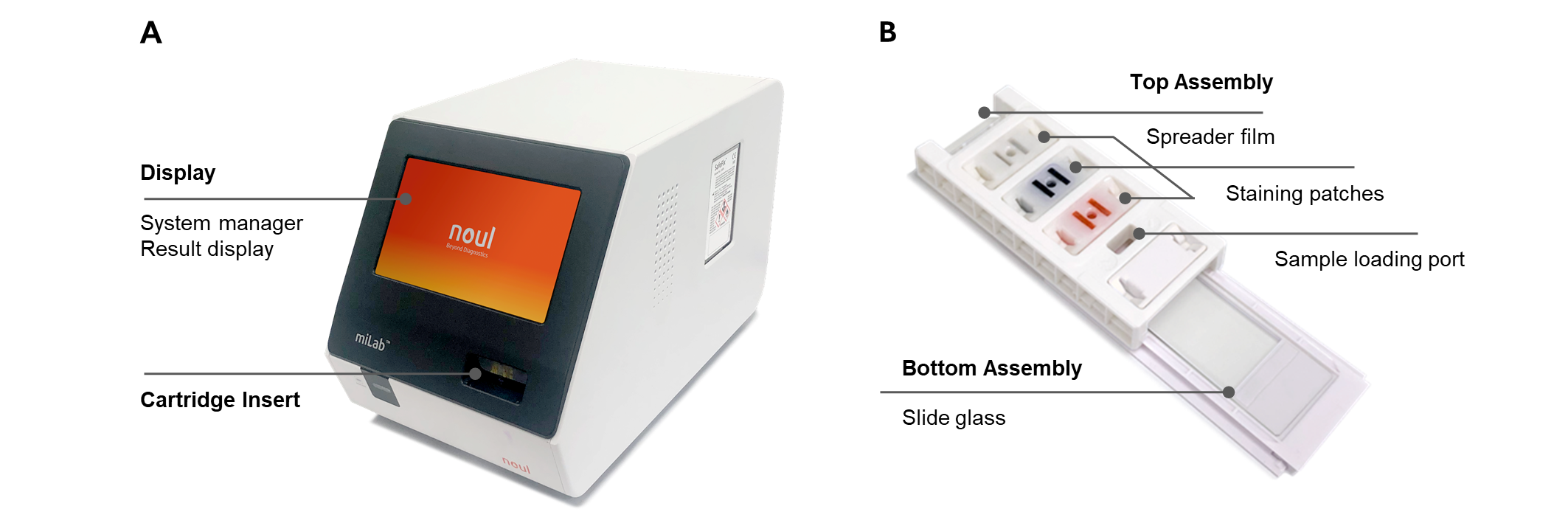

Supplement: Supplementary file 5 [file Image1.TIF]

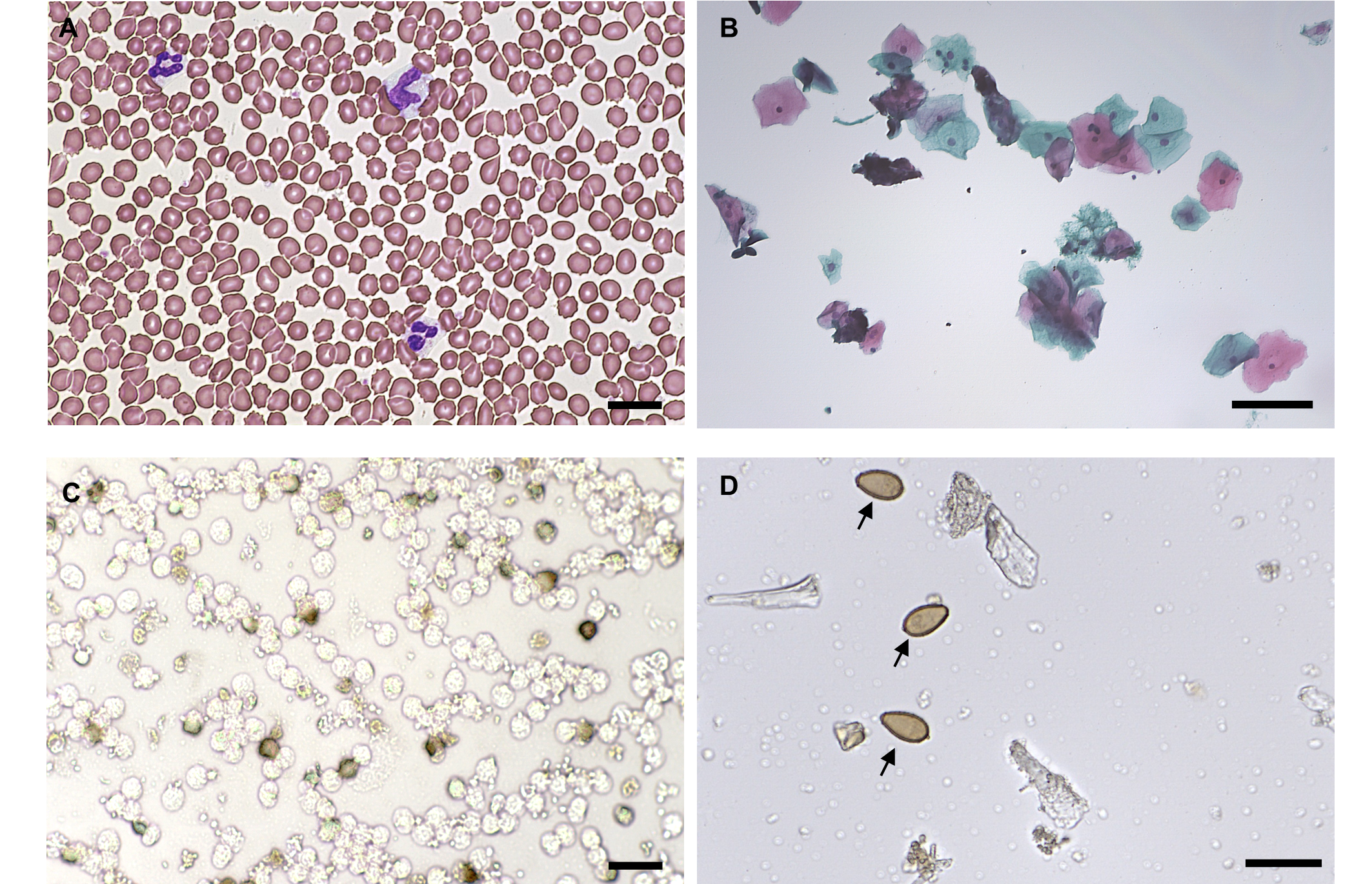

Supplement: Supplementary file 6 [file Image7.TIF]

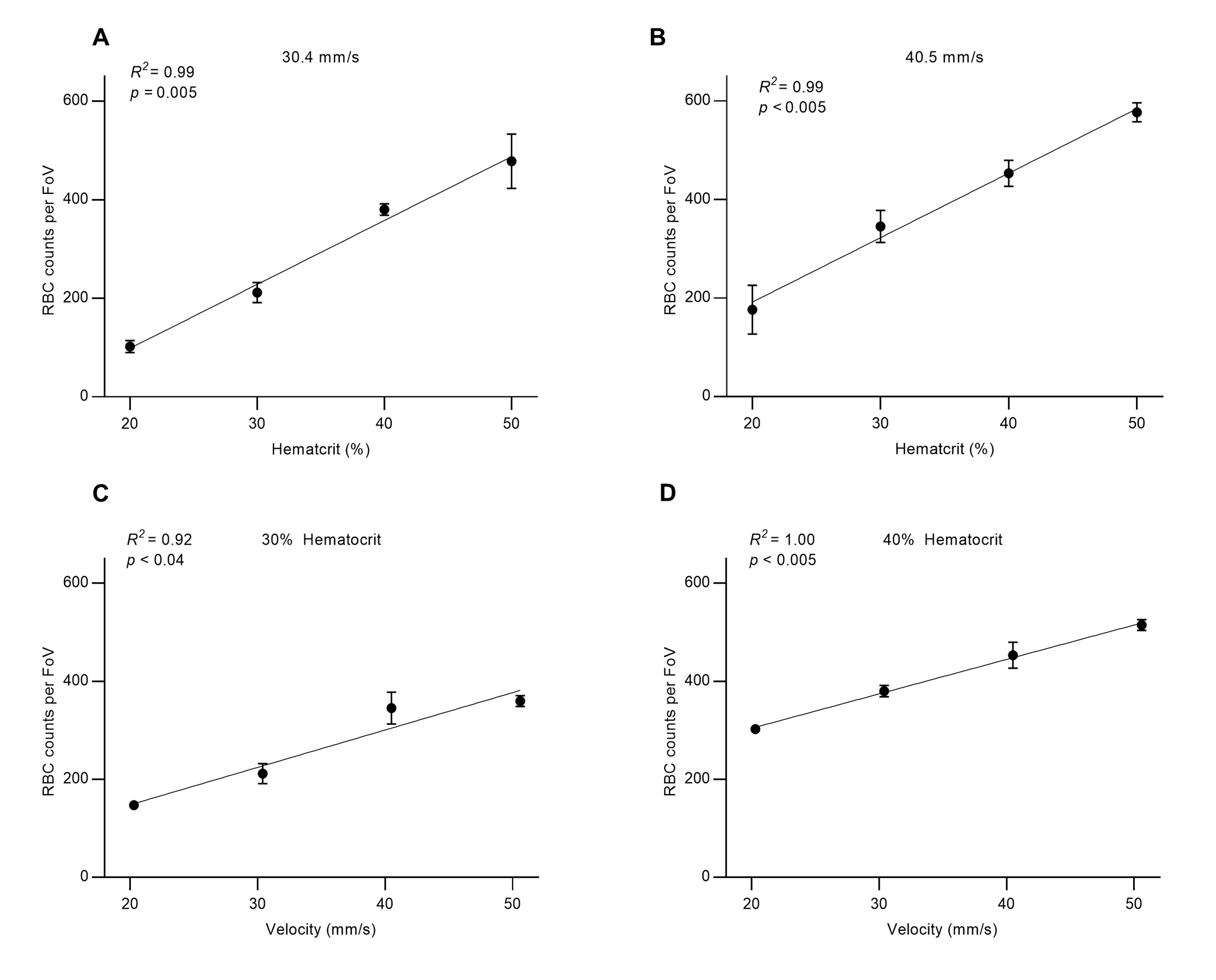

Supplement: Supplementary file 7 [file Image5.TIF]
